# Examining the Conservation of Kinks in Alpha Helices

**DOI:** 10.1371/journal.pone.0157553

**Published:** 2016-06-17

**Authors:** Eleanor C. Law, Henry R. Wilman, Sebastian Kelm, Jiye Shi, Charlotte M. Deane

**Affiliations:** 1 Department of Statistics, University of Oxford, Oxford, United Kingdom; 2 Department of Informatics, UCB Pharma, Slough, United Kingdom; 3 Shanghai Institute of Applied Physics, Chinese Academy of Sciences, Shanghai, China; University of Cambridge, UNITED KINGDOM

## Abstract

Kinks are a structural feature of alpha-helices and many are known to have functional roles. Kinks have previously tended to be defined in a binary fashion. In this paper we have deliberately moved towards defining them on a continuum, which given the unimodal distribution of kink angles is a better description. From this perspective, we examine the conservation of kinks in proteins. We find that kink angles are not generally a conserved property of homologs, pointing either to their not being functionally critical or to their function being related to conformational flexibility. In the latter case, the different structures of homologs are providing snapshots of different conformations. Sequence identity between homologous helices is informative in terms of kink conservation, but almost equally so is the sequence identity of residues in spatial proximity to the kink. In the specific case of proline, which is known to be prevalent in kinked helices, loss of a proline from a kinked helix often also results in the loss of a kink or reduction in its kink angle. We carried out a study of the seven transmembrane helices in the GPCR family and found that changes in kinks could be related both to subfamilies of GPCRs and also, in a particular subfamily, to the binding of agonists or antagonists. These results suggest conformational change upon receptor activation within the GPCR family. We also found correlation between kink angles in different helices, and the possibility of concerted motion could be investigated further by applying our method to molecular dynamics simulations. These observations reinforce the belief that helix kinks are key, functional, flexible points in structures.

## Introduction

Disruptions of the ideal helix geometry in proteins, often called kinks or bends, frequently occur in transmembrane helices (TMHs) and the long helices in soluble proteins [1]. At a kink, the helix axis changes direction and the helical hydrogen bonding pattern is often broken. Kinks are important as they have been shown to be flexible and/or carry out crucial functional roles, for example in G-protein coupled receptors [2].

Due to their importance, many methods have been developed to calculate kink angles in helices, including HELANAL [3], Prokink [4], MC-HELAN [5] and Kink Finder [1], and all define a kink differently. HELANAL and Kink Finder both define a kink as an angle of 20° or greater, however HELANAL defines a local helix axis vector using four residues while Kink Finder fits a cylinder over six residues. MC-HELAN locates all straight helical segments in a protein, and a helix is kinked if it comprises more than one segment. When the change of direction of helices is measured as an angle, the distribution is continuous [1], therefore there is not an obvious threshold angle above which helices are kinked. Even changes of direction of similar sizes can result from very different geometry and hydrogen bonding [4].

In an attempt to overcome this definition problem, an orthogonal approach was taken by Kneissl *et al*. [6], who manually assigned kinks. This was taken one step further by Wilman *et al*., who used crowd sourcing for kink identification, combining the observations of 310 people [7]. In most cases, there was no consensus on whether a helix was curved, kinked or straight, emphasising the difficulty of classification.

None of these methods, computational or manual, has any estimate of error on the kink angle measurements. Knowing the error on the kink calculation may help explain the discrepancies between definitions, and it will also allow us to test whether kinks in different structures are statistically different. In this work, we have developed a novel heuristic error estimation method, for use with the Kink Finder method.

Even with the difficulties in kink definition, particular residues have been repeatedly associated with kinks. The most important of these is proline, which often occurs in the residues following a kink (e.g. [1, 5, 8]). This is thought to be due to the lack of an amide hydrogen atom on the nitrogen atom of proline. In a helix, this atom would usually form a hydrogen bond to the backbone carbonyl oxygen of the residue four earlier. Proline is found in many of the helices with the largest kink angles, however, up to two-thirds of kinked helices do not contain proline (e.g. [5, 9]).

It has also been proposed that a proline could initially cause a kink to occur in a structure, after which it may mutate to another residue with the kink remaining [10]. This hypothesis suggests that kinks will generally be conserved despite changes in sequence, and that both local sequence effects and more global interactions with neighbouring helices should be considered. Another indicator that kinks may be a conserved feature is that kinks are often annotated as coil residues, and coil residues found in the central core of membrane helices (also including re-entrant helices) are often conserved [11].

The conservation of sequence and structure at the site of kinks indicates potential functional relevance. One important property of kinks for the function of proteins is their ability to create a flexible point within a structure. Molecular dynamics (MD) simulations on individual transmembrane helices [12] have shown a range of angles can be adopted by a single helix. MD on voltage-gated potassium channels [13] showed a range of conformations for their helices, which could be significant in ion channel gating. Betinelli *et al*. used a simplified model to explore conformational change in a large superfamily of membrane proteins: G-protein coupled receptors (GPCRs) [14]. They created conformational chimeras where the four proline-containing helices of GPCRs were independently able to adopt kinked or straight conformations, and the whole model was allowed to relax through MD simulations. They studied the stability of all 16 possible conformations and found that bound agonist favoured straight conformations, but bound antagonist favoured bent conformations. This suggests that kinks may have an important role in changes of conformation upon binding. This methodology was also applied to models of the human mAChR1 receptor, and used to improve virtual screening by using different conformations produced [15].

Flexibility of this type can also be seen by inspecting the differences between crystal structures of the same protein in different conformations. One case where this has been carried out is some of the subfamilies of GPCRs [16]. The inactive and activated structures of the *β*_2_-adrenergic receptor were compared and a ‘swinging’ and some unwinding of transmembrane helix (TMH) 6 was observed about the kink location [2, 16], but it was shown to maintain the same bend angle. Alpha bulges, also known as *π*-turns, are a feature of TMH 2 and 5 in most GPCRs, and these are points where twisting and bending is seen when comparing inactive and activated structures of other receptors [17]. HELANAL has been used to compare the kinks in inactive and activated rhodopsin, opsin and the *β*_2_-adrenergic receptor [18]. Differences in some of the kink angles were seen, however, as HELANAL has no method of estimating measurement error, the significance of these differences is difficult to evaluate.

These previous studies on kink flexibility mostly focus on one specific protein or model. In this paper, we compare helices from homologs, rather than from different structures of the same protein. This strategy makes it possible to use a much larger set of data. The differences in angles seen may be due to sequence differences between homologs, or they may be showing examples of kink flexibility. Using our new error estimation method, we are also able to test whether the angle differences are significant. We compare the angles of helices, without the need to classify them as “kinked” or “straight” according to an arbitrary threshold. We instead classify helices that display different angles as not conserved. We found that in sets of pairs and families of homologous proteins, it is common to find homologous helices which are differently kinked. We then carried out an extended analysis of the seven transmembrane helices of GPCRs. The kinks in TMH 6 and 7 are well conserved, but the others show greater variation. One receptor displayed a change of kink angle between agonist and antagonist bound structures, therefore our results also support the belief that kinks are functionally important.

## Methods

### Angle measurement by Kink Finder

Kink Finder [1] was used to measure angles in helices. Kink Finder fits a cylinder to every 6-residue segment of a helix by minimising *r*, where

$$\begin{array}{c} r=\sqrt{\frac{1}{m}\sum_{i=1}^{m}{\left(d_{i}-\bar{d}\right)}^{2}} \end{array}$$ (1)

*m* is the number of backbone atoms in the segment (24), *d*_*i*_ is the shortest distance from backbone atom *i* to the fitted helix axis, and $\bar{d}$ is the mean of all distances. The angle is measured between the axes of the cylinders fitted to adjacent segments, and this angle is assigned to the final residue of the first segment (Fig A in S1 Document). Only helices 12 residues or longer can be analysed.

### Method of confidence interval estimation

We have developed a method that estimates the error in each angle measured by Kink Finder. This heuristic method is based on observed errors in a set of ‘ideal’ kinks with good cylinder fits. Each cylinder fit has a value of *r* Eq (1), which measures the distance of the atoms from the cylinder surface. The calculation of a kink angle requires two cylinder fits, one for the set of six residues N-terminal of the kink position, and one for the set of six residues C-terminal to the kink (see Fig B in S1 Document). The goodness of these two fits (*r*_*n*_ and *r*_*c*_, the *r* for the N- and C-terminal cylinder fits) are assumed to have an equal effect on the angle error. For each measured angle, *θ*, the ‘goodness of fit’ is approximated by the sum of the *r* of the two cylinder fits. Although there is no way to directly measure the ‘true’ angle, 18 of the best fitted (‘ideal’) kinks were used to estimate the effect, assuming that the fitted axes for these provided the ‘true’ angle. These 18 helices (with the lowest *r*_*n*_ + *r*_*c*_, of the kinks in the membrane protein set) have a range of true angles between 0° and 50°. The *r*_*n*_ + *r*_*c*_ for all of these 18 is below 0.6 Å. Even for an ideal helix, *r*_*n*_ + *r*_*c*_ cannot be less than 0.27 Å.

Taking these ‘ideal’ kinks, we simulated the relationship between *r*_*n*_ + *r*_*c*_, *α* (the error), and the true angle. For each measurement in each kink, both cylinders were rotated about their midpoint by an angle and direction, using a randomly generated rotation matrix (Fig B in S1 Document). This provided a series of measured angles, *θ*, based on non-optimised cylinder fits. The *r*_*n*_ + *r*_*c*_ and *θ* were recorded for each, and used to characterise the relationship between the two (Fig C in S1 Document).

For a given range of *r*_*n*_ + *r*_*c*_, *α* (and similarly, *θ*), has a distribution that is close to normal. To find out how this distribution is related to *θ*, we assumed that it is normally distributed with mean zero and variance $\sigma_{\alpha }^{2}$:

$$\begin{array}{c} \alpha ∼N\left(0,\sigma_{\alpha }^{2}\right). \end{array}$$ (2)

The data was binned based on *r*_*n*_ + *r*_*c*_, and we made this assumption for each bin. In each bin for each kink, *σ*_*α*_ was calculated. Excluding kinks under 10°, the size of *α* can be considered to depend only on the value of *r*_*n*_ + *r*_*c*_ (Fig D in S1 Document). Therefore, the errors for all of the kinks with angles above 10° were combined to calculate the relationship between *r*_*n*_ + *r*_*c*_ and angle error.

From this point, a statistical confidence interval was used, rather than using the standard deviation, as this does not rely on the assumption of normality. From the 12 kinks over 10°, all values of *α* were binned into ranges of *r*_*n*_ + *r*_*c*_. The distribution is symmetric, and is assumed to be so in the rest of this work. The 95th percentile of |*α*| for each bin was taken to give *ε*, deriving the size of the statistical confidence interval:

$$\begin{array}{c} \text{95\% confidence interval for true angle}=\theta \pm \varepsilon \end{array}$$ (3)

where *θ* is the measured angle, and we will refer to *ε* as the error. We use a log fit to quantify the relationship between *r*_*n*_ + *r*_*c*_ and *ε* (Fig E in S1 Document), which gives:

$$\begin{array}{c} \varepsilon =\left(6.349\times \text{ln}(r_{n}+r_{c}-0.2937\right)+13.15) \end{array}$$ (4)

Thus *ε* is an estimate of the uncertainty in the kink angles measured by Kink Finder and provides a simple way to compare the angles in two helices. Kink Finder is available online at http://www.stats.ox.ac.uk/research/proteins/resources.

### Data sets

We gathered sets of soluble and membrane protein chains using the same methodology as in [1]. Soluble protein chains were obtained on 3rd March 2015 by filtering the protein data bank (PDB) [19] using PISCES [20] to select chains with <80% sequence identity to each other, resolution <5 Å, 40 < chain length < 1000 residues, and R-factor <0.4. The set includes the first conformer of NMR structures but structures from electron microscopy experiments, and C*α* atom-only structures were removed. Any protein chains in the PDBTM [21], Membrane Proteins of Known Structure Database [22] or Orientations of Proteins in Membranes database [23] were removed to eliminate transmembrane structures. A second set of soluble proteins was obtained in the same way, but containing crystal structures only and with resolution <2 Å and R-factor <0.2. Table A in S1 Document shows the results for this high quality dataset.

To create a set of membrane protein chains, a list of polytopic alpha-helical membrane protein PDB codes was taken from the Membrane Proteins of Known Structure Database on the 8th January 2015 and the PDBTM on 9th January 2015. After splitting into chains, a clean non-redundant set was generated as described above for the soluble set. A redundant set of membrane protein chains was also kept where maximum sequence identity was 99%.

The G-protein coupled receptors database (GPCRDB) [24], accessed 4 November 2014, provided a set of 122 chains from crystal structures of G-protein coupled receptors of 28 different receptors. The database provides its own numbering scheme and structural alignment for these receptors.

For helices from each data set, the distribution of maximum angles measured by Kink Finder is shown in Fig F in S1 Document. A list of PDB codes in each set is available in S1 Data.

#### Identifying homologous helices

For the sets of soluble, membrane, and redundant membrane chains, helices were annotated using JOY [25]. Any helical segments separated by only one or two residues were combined. The ends of helices were trimmed until they satisfied the criteria for a helical seed used by MC-HELAN [5]. For membrane proteins, helices were only kept if at least one residue was annotated to be in the tail region of the membrane by iMembrane [26].

An all-against-all structural comparison of the protein chains in a set was carried out, using TM-align [27]. Pairs of helices were considered homologous if:

- the number of residues in the longer chain was no more than 50% greater than the number in the shorter chain
- the two structures shared the same fold, indicated by a TM-score of the alignment greater than 0.5 [27]
- the sequence identity in the TM-align alignment was at least 10% (ignoring gaps)
- the two helices aligned in such globally similar structures had ends that were offset by no more than four residues in the TM-align alignment

This resulted in 629,524 homologous helix pairs in soluble chains (SolPairs), 4,104 pairs in the membrane chains (MemPairs), and 41,945 pairs in the redundant set of membrane chains (RMemPairs).

#### Identifying homologous aligned families of helices

Using the non-redundant membrane homologous helix pairs, a network of helices was constructed, where an edge connected each pair of homologous helices (defined above). Communities of related helices were extracted using the software of [28], with resolution parameter *λ* = 25. All members of a community had to be connected to >90% of the other members in order to be a helix family. If a community had any member with connectivity <90%, the member with the lowest connectivity was removed. Connectivity of other members was recalculated and the process repeated until all members satisfied the requirement of connectivity >90%. Families of fewer than five members were discarded, leaving 45 membrane helix families (MemFams). This process was repeated for soluble helices and 1258 soluble helix families (SolFams) were identified. For each helix family, a multiple structural alignment of the full protein chains was generated using MAMMOTH-Mult [29].

#### Obtaining helix families for the seven transmembrane helices (TMHs) of G-protein coupled receptor structures (GPCRs)

The GPCRDB provides a structure-based alignment of each of the helices of GPCRs [24]. The sequences in GPCRDB are the native sequences, but some GPCR structures contain mutations. In order to align these structures correctly, they were aligned to the receptor sequence provided without introducing any gaps. The GPCRDB alignments of helices were truncated to the first and last residues in each helix where more than half of the structures did not have a gap. TMH 7 was further shortened to start from residue 7x33, as the consensus secondary structure annotated by JOY [25] for the first four residues was not helical. This resulted in a helix family of at least 113 members for each of the GPCR TMHs.

### Comparison of two helices using error estimation

**Fig 1:**
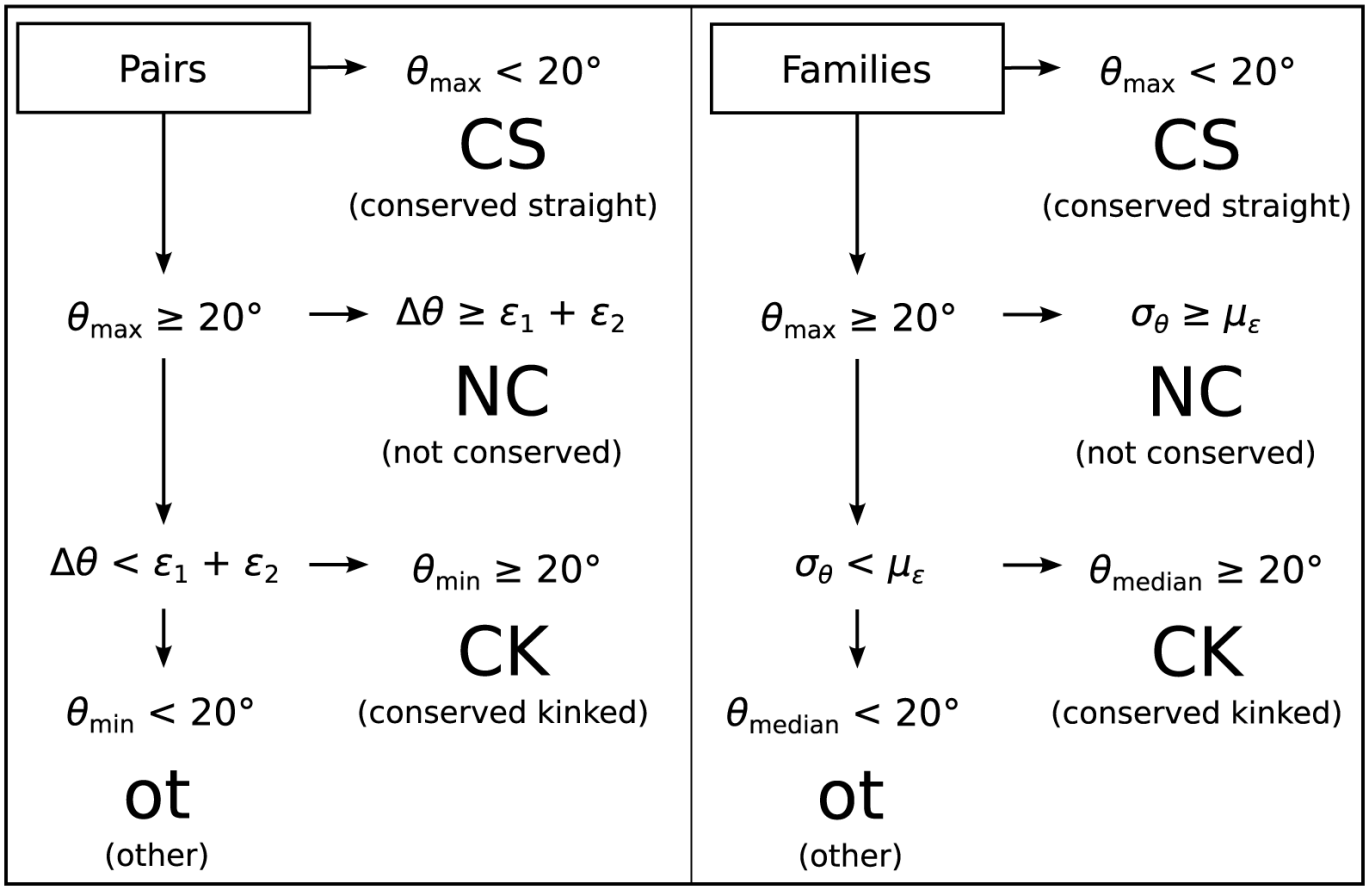
Flowchart showing the classification of homologous helix pairs and families. Pair classification uses the angles of the two helices at the most disrupted site (*θ*_max_, *θ*_min_), angle difference (Δ*θ*) and the error on each angle (*ϵ*). Family classification uses analogous statistics to obtain the same classes: the median angle (*θ*_median_), standard deviation (*σ*_*θ*_), and mean error (*μ*_*ϵ*_) of the angles in the family.

Kink Finder (described above) was used to measure angles at all sites in all of the helices in our sets. For each homologous helix pair, the site of the greatest angle in either helix was used as the most disrupted site for classification. This angle was compared to the largest angle in the other helix, within a window of ±4 residues either side of the kink site. A window was used because it is both difficult to accurately define the position of a kink, and there may also be error in the alignment. If no angle was found in this window due to gaps, the helix pair was removed from the set. The pairs were then classified by the scheme shown in Fig 1 using the error estimate on each angle, *ε* Eq (4). The “Not Conserved” class includes homologous helix pairs where both helices are kinked, but with significantly different angles.

When the difference between two angles is less than the sum of the errors on those angles, the difference is not significant. In these cases there may be a real difference between the angles but it is not found to be significant. This could lead to an underestimation of the number of kinks which are not conserved, classifying some as Conserved Straight, Conserved Kinked or Other.

#### Calculation of ‘neighbouring sequence identity’

We calcluate the ‘neighbouring sequence identity’ of a homologous helix pair as a measure of sequence conservation among the residues in spatial proximity to the helices. For every helix in the data set, we found all surrounding residues in the chain which had at least one atom within 4 Å of an atom in the helix. For a pair of homologous helices, the ‘neighbouring sequence identity’ is the sequence identity over all the positions which were in the set of neighbouring residues for either helix and which were not gaps in the structural alignment.

### Classification of families based on a ‘most disrupted’ site

For the homologous helix families in the MemFam and SolFam sets, the MAMMOTH-Mult alignment was used to compare helices; for the GPCR set, the GPCRDB alignment was used. Angles were measured by Kink Finder at each residue, and assigned to that residue’s position in the alignment. For each residue in a helix in the alignment, the maximum angle from a window of three residues was used as the smoothed angle for that residue (Fig G in S1 Document). The smoothing allows for inaccurate alignment when comparing the angles around the sites of the largest angles. The angles of each of the helices in a family can then be compared at every site in the alignment.

Only one site of maximum disruption was used to classify a family. The site in the helix with the highest mean was chosen as the most disrupted site. Only sites in the alignment of the angle data which had at least five recorded angles after smoothing were considered. To determine the variation of angles at the most disrupted site, standard deviation was calculated. The standard deviation was compared to the mean error of angles at the most disrupted site in order to classify families as conserved or not. The flowchart in Fig 1 shows the method of classification for helix families.

A package containing all data for the homologous helix pairs and families is available in S1 Data.

## Results

### Confidence intervals of angles measured by Kink Finder

**Fig 2:**
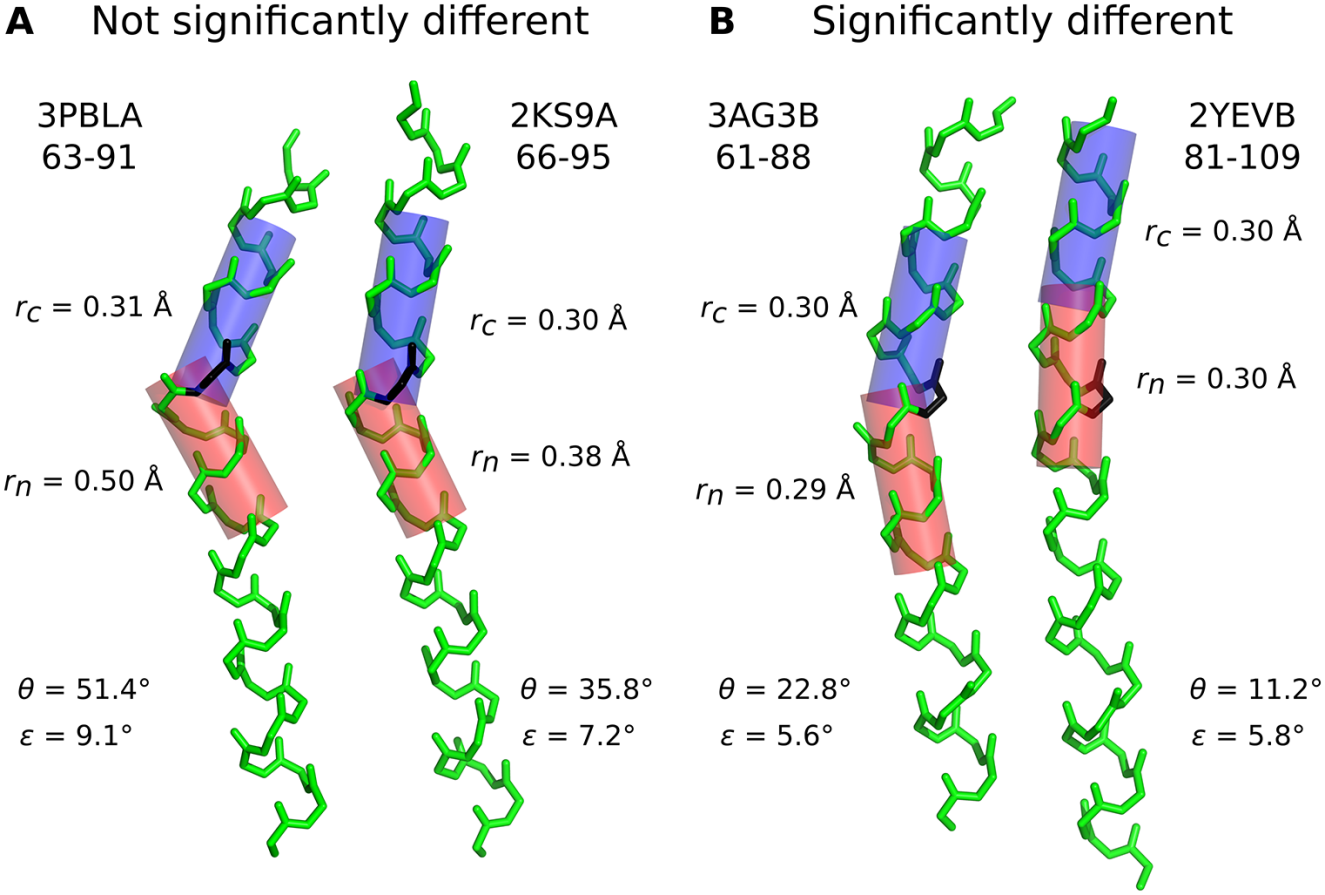
Two examples of helix pairs, which are (A) not significantly different and (B) significantly different. PDB code, chain identifier and residue numbers are given for each helix. The black residues are at the most disrupted site (see Methods) in each helix pair. *r*_*n*_ and *r*_*c*_ give the quality of the cylinder fit (see Eq (1)) to the backbone atoms on the N- (red) and C- (blue) terminal sides of the kink site. *θ* is the angle measured between the two cylinders. *ε* is the estimated error of the angle measurement, calculated from *r*_*n*_ + *r*_*c*_ using Eq (4). If *θ*_max_ − *θ*_min_ > *ε*_1_ + *ε*_2_, the confidence intervals do not overlap therefore we consider the angles to be significantly different.

In this paper we analyse whether helix kinks are conserved between homologs by comparing their angles. The first step is to calculate a confidence interval on the helix kink angles measured. We used our novel method within Kink Finder [1] to calculate the confidence interval for every angle (see Methods). The typical range of error sizes is around 5-8°, and larger kinks are generally associated with larger errors (Fig H in S1 Document), due to poorer fits. Fig 2A shows two helices which have a difference in angle of 15.6°. However, we cannot be sure in this case that the kink angles are different because the quality of fit is poor, and therefore the 95% confidence intervals (*θ*±error, *ε*) are overlapping. In Fig 2B, two helices are shown that have angles which differ by only 11.6° but the confidence intervals do not overlap because the quality of fit is better. Thus we consider these helices to have significantly different kink angles.

### Homologous helix pairs

#### Number of helix pairs found

Non-redundant sets of 18,934 soluble and 392 membrane protein chains with at least one helix of 12 or more residues were collected. From these, 4,104 aligned pairs of homologous membrane helices (MemPairs) and 629,524 aligned homologous soluble helix pairs (SolPairs) were extracted. Kink Finder was used to measure the angles and the error on these angles for all residues in every helix.

#### Definition of pair classes

The homologous helix pairs were divided into four classes, based on the size and uncertainty of the kink angles in the two helices (Fig 1):

**Conserved Straight** *θ*_max_ < 20°
**Conserved Kinked: no significant angle variation** *θ*_min_ > 20°, *θ*_max_ − *θ*_min_ < *ε*_1_ + *ε*_2_
**Not Conserved: significant angle variation** *θ*_max_ > 20°, *θ*_max_ − *θ*_min_ > *ε*_1_ + *ε*_2_
**Other** All other pairs

*θ*_max_ and *θ*_min_ are the larger and smaller of the measured angles in the helix pair; *ε*_1_ and *ε*_2_ are the errors of the two angles. The number of helix pairs in each of these classes is shown in Table 1. Conserved Straight (CS) is the most common class for both soluble and membrane proteins, with 77% of soluble helix pairs and 44% of membrane helix pairs belonging to this class. As expected, kinks are more common in the membrane protein set, probably because membrane helices are longer and longer helices are more frequently kinked [1]. Conserved Kinked (CK) pairs are more frequent than Not Conserved (NC) pairs in the membrane set (29% compared to 19%) but soluble proteins show the opposite trend (6.4% compared to 14.0%).

**Table 1:** The number of aligned helix pairs in each class, and occurrence of proline within that class.

	Membrane					Soluble				
	CK	CS	NC	other	total	CK	CS	NC	other	total
All	1189	1806	789	320	4104	40190	481388	88390	19556	629524
%	29.0	44.0	19.2	7.8	100.0	6.4	76.5	14.0	3.1	100.0
PP	14.4	0.9	1.9	1.8	19.0	1.3	0.0	0.4	0.1	1.8
P-	4.1	2.7	7.7	1.1	15.6	0.9	0.5	5.7	0.4	7.5
-P	4.4	0.2	1.3	0.8	6.7	0.8	0.1	0.3	0.0	1.2
- -	6.1	40.2	8.4	4.1	58.7	3.3	75.9	7.7	2.6	89.5

The helix pair classes conserved kinked (CK), conserved straight (CS), not conserved (NC) and other are defined in Fig 1. The frequency of proline in each class is given as a percentage of the total number of pairs. PP: proline in both helices; P-: proline in the helix with the larger kink angle; -P: proline in the helix with the smaller kink angle; - -: proline in neither helix. All percentages are rounded to one decimal place.

#### Presence of proline in kink pairs

We tested whether the four classes are different in terms of proline occurrence, as proline is commonly associated with kinks. Proline was identified as present if it was at the position of the largest angle or in the four following residues. Table 1 shows the presence of proline in the aligned helix pairs, broken down by pair type (for a detailed breakdown see Table A in S1 Document).

Proline is common at the most disrupted site in membrane helix pairs, occurring in both helices in 19% of pairs, however it is much less common in soluble pairs and more often present in only one of the two helices (7.5 + 1.2 = 8.7%) rather than in both (1.8%). For both membrane and soluble proteins, when proline is present in both helices, most pairs are Conserved Kinked. When proline is not present in either helix at the most disrupted site, Conserved Straight is most common. In the case where proline is present in a kinked helix but not conserved in its homologs, it has been suggested that the kink will be conserved throughout the family. However, in our data, if a helix has a proline and is kinked and its partner helix does not have a proline, the pair may be Conserved Kinked or Not Conserved. Not Conserved is nearly as common as Conserved Kinked (when P- and -P are combined) for membrane helix pairs, and more common in soluble helix pairs, indicating that loss of proline is often accompanied by a significant reduction in kink angle.

### Relationship between angle difference and sequence identity

**Fig 3:**
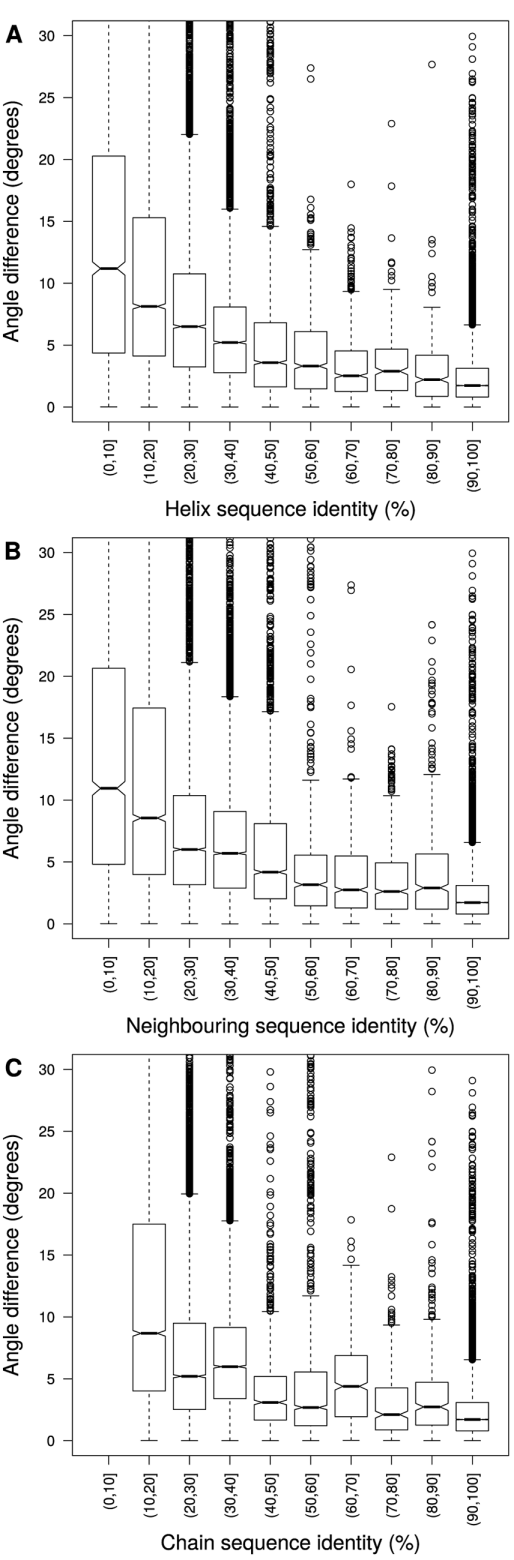
The difference in angle at the most disrupted site between helices in homologous helix pairs (|*θ*_max_ − *θ*_min_|) plotted against the sequence identity between A) the homologous helix sequences, B) the residues in spatial proximity to the homologous helices (neighbouring residues) and C) the homologous chain sequences. Data from the redundant membrane protein set is shown so that the full range of sequence identity can be seen.

As we are considering the angle difference between homologous helices, we wanted to investigate how it relates to the sequence identity between helices (Fig 3A), the sequence identity between neighbouring residues (Fig 3B, calculated as described in Methods), and the sequence identity between the complete chains (Fig 3C), in all cases ignoring gaps. As expected, there is a trend for larger angle differences to be associated with lower sequence identity. Conversely, kinks are well conserved when sequence is conserved, whether on a local or global scale. For the non-redundant membrane set, the Spearman’s rank correlation coefficients are very similar for Helix (-0.28), Neighbouring (-0.29) and Chain (-0.27) sequence identity. The partial correlation coefficients, when using other measures of sequence identity as controlling variables (see Table B in S1 Document) are also similar to each other (∼−0.1), but show that each factor makes an independent contribution to kink angle changes. This is also true for our non-redundant set of soluble proteins, but the correlation coefficients are weaker (∼−0.1).

### Homologous helix families

#### Number of families found

As homologous helix pairs showed large numbers of unconserved kinks, we built families of homologous helices in order to investigate kink conservation patterns across larger samples of related helices. Families contained at least five members, and Table 2 gives the number of families of various sizes.

**Table 2:** The number of helix families greater than or equal to each group size for the soluble and membrane protein sets.

Group size	5	10	20	50	100
Membrane	45	18	0	0	0
Soluble	1258	473	170	28	4

#### Definition of family classes

**Fig 4:**
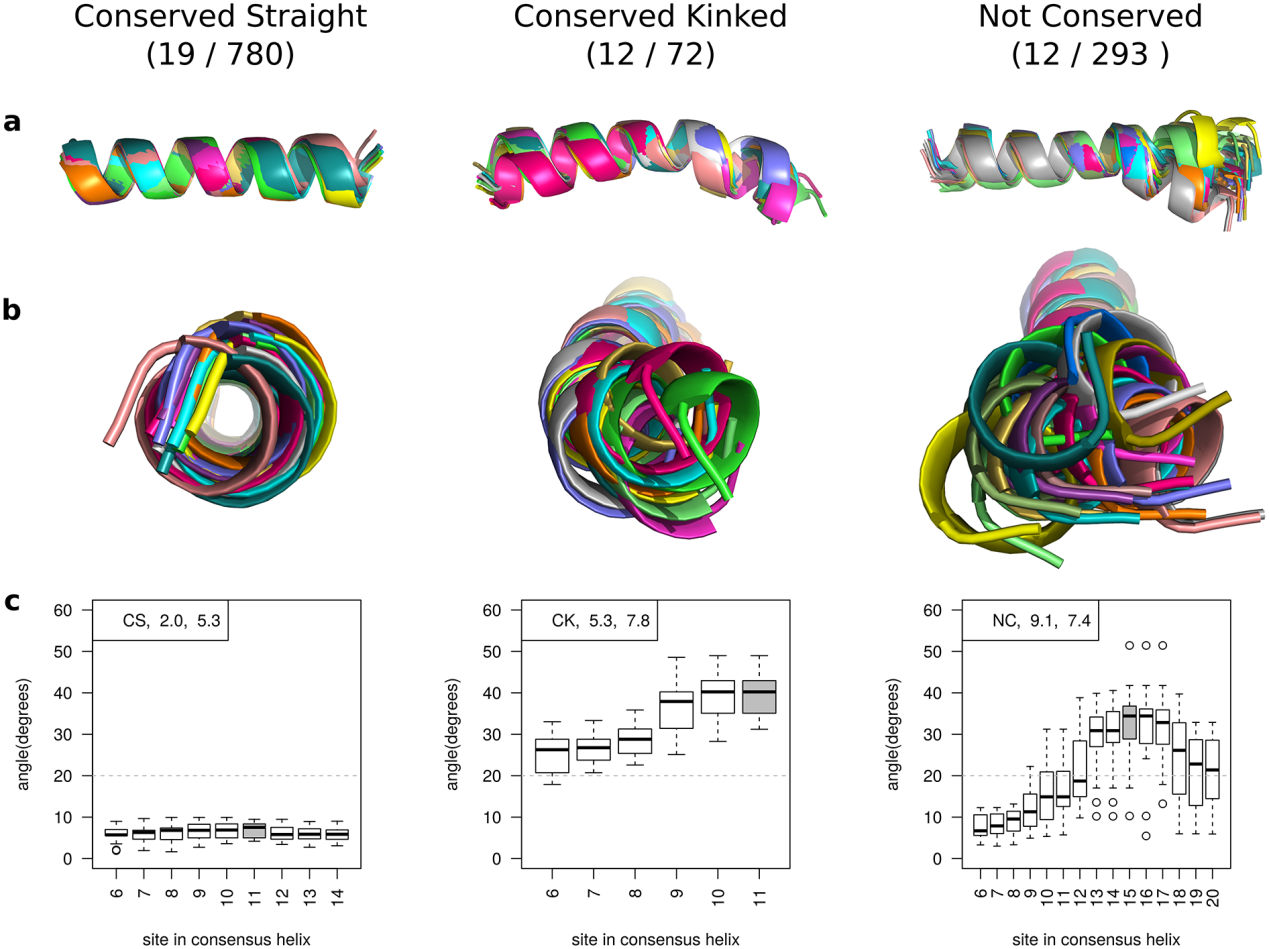
Illustrations of a homologous helix family from each of the three main classes. The number of families in each class is shown in brackets (membrane set / soluble set). 113 out of 1,258 soluble families and 2 out of 45 membrane families were classified as ‘Other’. **(a)** and **(b)** Side and top view of helices in a family superimposed by aligning the residues prior to the kink site. **(c)** Boxplots to show the variation in angle after smoothing at each site in the helix. The grey box indicates the site of maximum disruption used to classify the helix (see Methods). In the top left of each graph, the classification, *σ*_*θ*_ (standard deviation of angles), and *μ*_*ε*_ (mean error) of the most disrupted site is given.

Angles were measured for all members in a family and smoothed as described in the Methods. Each family was classified in an analogous way to the pair classification (Fig 1). Fig 4 shows an example of one family from each class. As with homologous helix pairs, Conserved Straight families are the most common class for both membrane and soluble families (19/45 and 780/1258 respectively). In soluble proteins, Conserved Kinked families are rare (72) compared to Not Conserved families (293), but in membrane proteins they are equally common (12 of each).

#### Prevalence of proline in different family classes

The relationship between prolines and kinks in our homologous helix families gives similar patterns to those seen for pairs (Fig L in S1 Document). If proline is found at the most disrupted site or the four following residues in every member of a family, it is a good indicator of kink conservation, as it was for helix pairs. Of the 10 membrane and soluble families where proline is fully conserved, 2 are classified as not conserved. For the 267 membrane and soluble helix families where proline is present in some but not all members, families are more frequently Not Conserved (175) than Conserved Kinked (43). Thus, once again proline conservation does not equal kink conservation in every case, and proline loss may or may not equal kink loss.

#### Relationship between angle variation and sequence conservation

In an analogous way to that used for pairs, we analysed the relationship between kink angle conservation and sequence conservation. We calculated the chain “sequence identity” for a family as the mean sequence identity between every pair of chains in the MAMMOTH-Mult alignment, ignoring gaps. Helix sequence identity was calculated in the same way using just the consensus helix positions. The relationship between family angle variation and sequence conservation (Fig J in S1 Document) is similar to that seen for pairs (Fig 3). There are very few data points at the higher end of the sequence identity range, even when combining all soluble and membrane data, as the family detection method led to most families containing at least some distant homologs. The partial correlation coefficient for helix sequence identity, given chain sequence identity as a controlling variable, is -0.16 and that for chain sequence identity, given helix sequence identity, is -0.11. This reinforces the suggestion found with the pairs that local and global sequence changes are both associated with kink angle changes.

### G-protein coupled receptor case study

**Fig 5:**
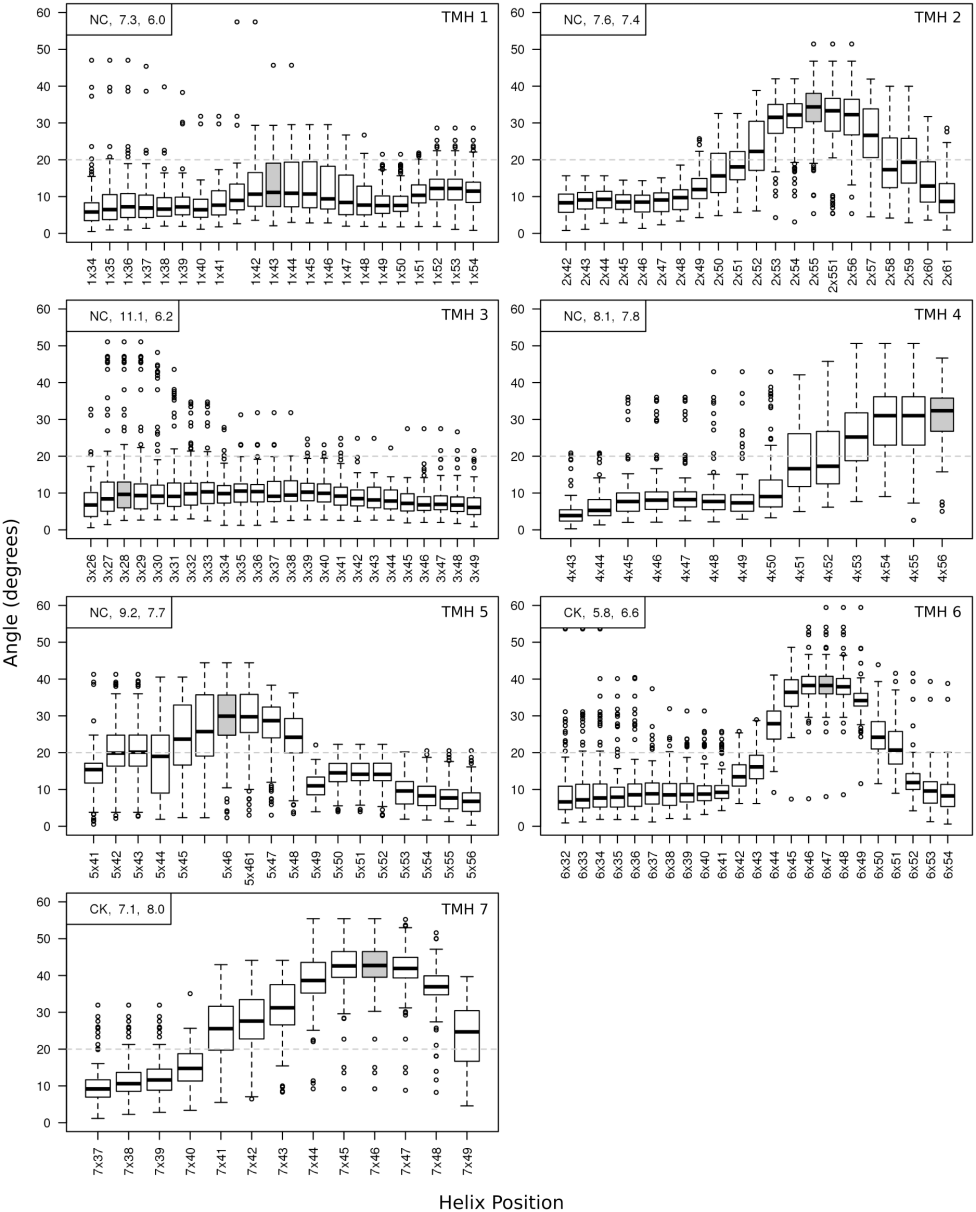
Distributions of angles measured at each site of the seven transmembrane helices in the GPCR family, after smoothing. The label at each site shown on the *x*-axis is the Class A numbering used in the GPCRDB [24]. The broken grey line at 20° is the threshold for the definition of a kink. The most disrupted site in each helix (see Methods) is shown in grey. In the top left of each graph, the classification, *σ*_*θ*_ (standard deviation of angles), and *μ*_*ε*_ (mean error) of the most disrupted site is given.

As a specific application of our methodology, we have carried out a detailed study of the transmembrane helices (TMHs) of GPCRs. The kinks in some GPCR helices are thought to be important for function [2], and many GPCR structures are available [30]. The structural alignment from the GPCRDB [24] was used to construct the smoothed angle profiles shown in Fig 5. The most disrupted site in each helix (see Methods) is shown in grey, and the standard deviation and classification of each helix is given.

**Fig 6:**
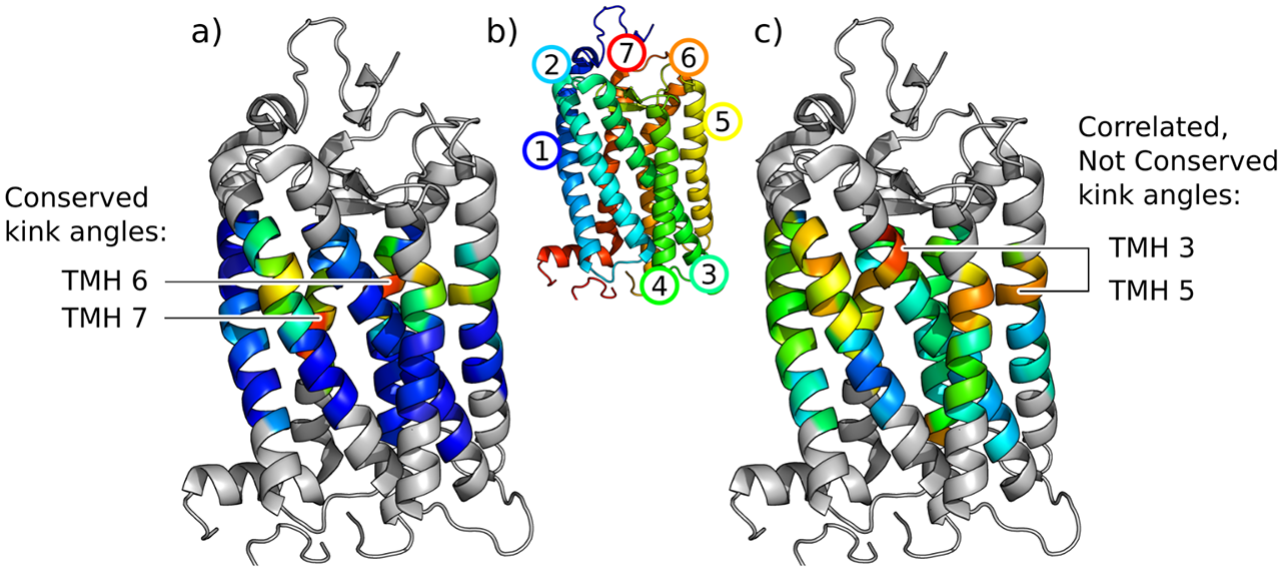
GPCR kink angle variation shown on the PDB structure 1F88, chain A. Colouring is by a) mean and c) standard deviation of angles at each site in the GPCR family, on a spectrum from the lowest values in blue to the highest in red. Grey residues have no angles measured as they are loop regions or within 6 residues of the end of the consensus helix (the minimum for a cylinder fit). b) is coloured by rainbow from N-terminus (blue) to C-terminus (red). The conserved kink angles in TMH 6 and TMH 7 and the correlated kink angles at sites in TMH 3 and TMH 5 are labelled.

TMH 1 is generally straight, but displays a wide variation in kink angles around residue 1x43 (Class A numbering system from the GPCRDB, based on structural alignment). TMH 2 has a kink at residue 2x55 which is present in almost all members, but shows a wide range of angles and is therefore classified as Not Conserved. TMH 3 is straight for almost all members of the GPCR family, however there is a small group which show a kink angle of up to 50° at site 3x28. TMHs 4 and 5 have a kink in most members at the most disrupted site, but like TMH 2, these kinks take a wide range of angles. TMH 6 and 7 are the only helices which have a kink classified as conserved. They also have the largest kink angles (Fig 6a). In TMH 6, only one member does not have a kink at position 6x47, and most other members are tightly grouped around 40°, with a standard deviation across the family of 5.8°. There also seems to be a not conserved kink present in a small number of members around residue 6x34, near the N-terminal end of the helix. The kink in TMH 7 at 7x46 shows a similar profile to TMH 6, however the standard deviation is slightly higher at 7.1°, though this is still less than the average error in this family of 8.0°.

Fig 6 shows the average size of angles measured at each site and their conservation across the family, presented on a structure of rhodopsin.

#### Angle variation relationship to sequence or flexibility

**Fig 7:**
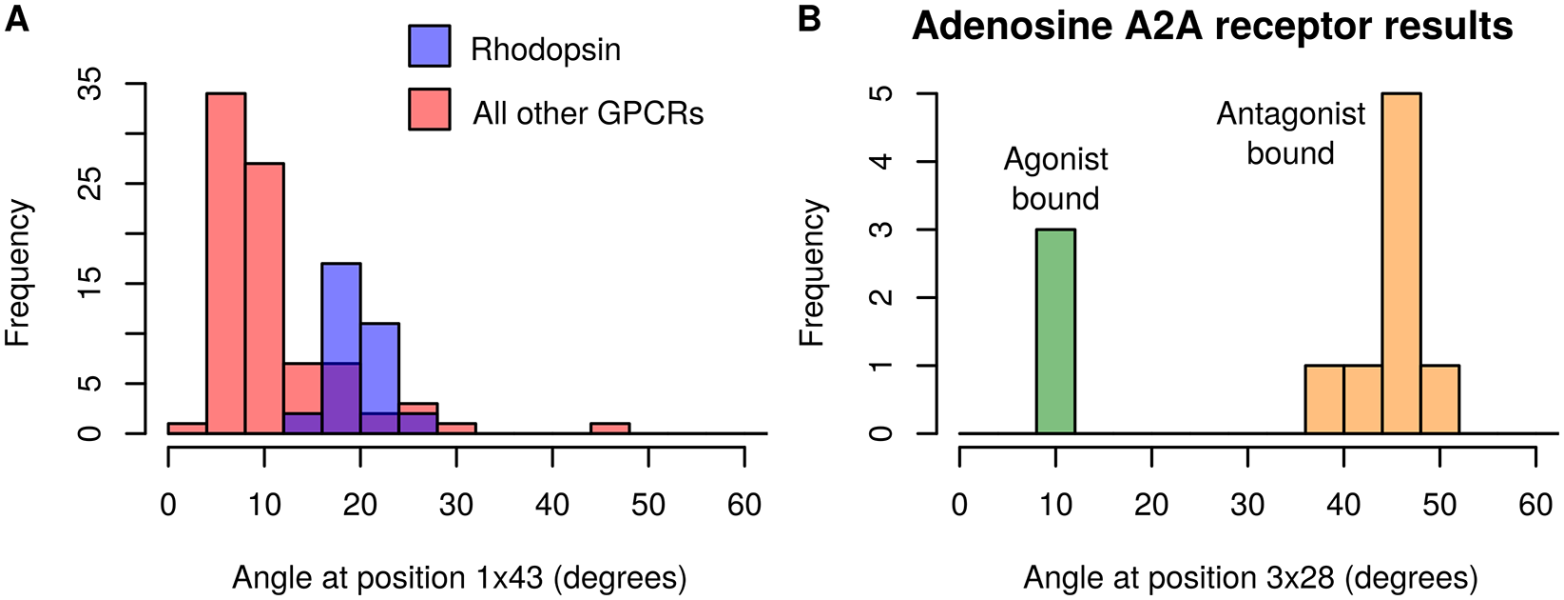
Bimodal angle distributions. **A)** Angle distribution at position 1x43 in all GPCR structures. Angles from rhodopsin structures are shown in blue (n = 32); angles from all other structures shown in red (n = 83). **B)** Angle distribution at position 3x28 in the human adenosine A_2A_ receptor. Agonist-bound receptors are shown in green (n = 3); antagonist-bound receptors in orange (n = 8). Fig M in S1 Document displays the errors for the angle data from both histograms.

In the GPCR set, multiple structures were available for some receptors. This made it possible to observe variation in angles for an individual receptor. We could also compare the distribution of angles for one receptor to the distributions of others. For example, in the not conserved TMH 1 at residue 1x43, we observed that the distribution of angles for rhodopsin was separated from most other GPCRs (Fig 7A, Fig N in S1 Document for all seven helices). The kink at 1x43 is in a functionally significant region and present in just a few GPCRs [31]. Rhodopsin has a proline at residue 1x48, and in 30 of the 32 rhodopsin structures in the set, the angle at 1x43 is >16°. There are 14 structures of 8 other GPCRs which also have angles >16°. Three of these GPCRs have proline near the kink, but there is no obvious sequence causing the other five to be kinked. Proline is not present near this location in any of the non-rhodopsin structures with angles <16°.

The full set of GPCR structures was also separated based on the type of ligand bound, but there was no difference between agonist, antagonist and inverse agonist structures overall. However, there were four receptors for which at least ten structures were available in the GPCRDB, so comparisons could be made between the different activation states of these individual receptors. One of these, the human adenosine A_2A_ receptor, displayed an angle change of over 30° at the most disrupted site in TMH 3. This site has the highest standard deviation of any angle in any of the seven GPCR helices (coloured red in Fig 6c). In the case of the A_2A_ receptor, the angle change is from straight in the agonist-bound structures to kinked in the antagonist-bound structures (Fig 7B). This suggests that the change in angle is involved in the conformational change which occurs on activation of the receptor. The kink location in the helix is at the binding site for the natural ligand, therefore in this case its flexibility seems to be particularly important for the function of the receptor. This change of helix shape has previously been described qualitatively [32].

#### Correlation between kink angles

Our method also allows us to identify correlations between the kink angles in different helices. These could suggest concerted motion or interaction between the helices, where the kinking of one helix affects the structure of the other.

An example in GPCRs is TMH 3 and TMH 5, where the Spearman’s rank correlation coefficient is 0.68 (Fig O in S1 Document). The sites of these kinks are slightly separated in the structure with TMH 4 between them (Fig 6c). There is a weaker correlation between the angle at these sites and the TMH 4 kink: 0.31 for TMH 3/4 and -0.35 for TMH 4/5. These correlations suggest that change of conformation in one helix can influence the conformation of another.

## Discussion

Using Kink Finder, we have developed a new method of error estimation which makes it possible to compare helices and state whether their angles are different. The method, which is based on the quality of fit to the helix either side of the kink, is to our knowledge the first that is able to obtain a confidence interval on a measured kink angle. Estimated errors are usually between 5–8°, and tend to be slightly larger for larger kink angles.

Kink Finder uses a method of fitting cylinders to either side of the kink, which has the advantage of reliably finding changes of direction even in the presence of non-canonical hydrogen bonding regions. This method requires a helix of 12 residues or more, but it is known that longer helices are more frequently kinked [1]. This results in a single metric, the kink angle, which allowed us to understand the error distribution and state the statistical significance of a difference in angles. There are other aspects of kink geometry such as the swivel angle which may not be conserved between our “conserved kinked” helices, therefore it is likely that we overstate kink conservation.

In this work, for an overview of kink conservation, we chose to classify each helix pair or family using one most disrupted site. This avoids biasing the results, however it represents a simplification of the more detailed information shown in Fig 5. As we show for GPCRs, analysing all positions in a helix of interest reveals more about the system. In order to facilitate such analysis, this data is available for all of our helix families (S1 Data).

Using the error estimation method, we have shown that kinks are not always conserved across structural homologs, i.e. they have significantly different angles. The different conformations seen in a pair or family can be explained by two possibilities, or a combination of both:

- The differences in sequence between two related proteins causes them to adopt different conformations.
- There is conformational flexibility at the kink, which could be important for function.

An investigation of the sequence dependence of the changes in angle provides evidence in support of the first option. The exact sequence drivers for kinks remain elusive. Even the loss of proline, the residue most closely associated with kinks, is associated with a significant reduction in kink angle in only 4/9 cases. Changes in conformation would be important to understand when modelling a homolog with no proline where the template has a proline kink, or vice versa. More generally, changes in angle are associated with changes in sequence. We found that this relationship between kink conservation and sequence conservation is similar, whether considering only the residues in the homologous helices themselves, the residues in spatial proximity to the homologous helices, or the complete chains of the homologous proteins. Current kink modelling approaches assume that global effects are important for kink formation, especially for predicting the size of kinks [33]. At the same time, sequence predictors make accurate kink predictions based on the primary sequence of the helix alone [5, 34]. Our results suggest that both local and global factors are indeed independently important, but that one usually accompanies the other.

In our in-depth analysis of GPCRs, we also found evidence that kinks can significantly change conformation within a single protein. In the case of the human adenosine A_2A_ receptor, there were multiple structures in our set, some with antagonists bound and others with agonists. Previous analysis of these structures reported a change in TMH 3 from kinked to straight between the two states [32, 35, 36]. A rearrangement of water molecules in the binding site is associated with the conformational change [32]. We quantify the change from kinked to straight as an angle change of 30° and we verify the statistical significance of the difference between the inactivated and activated conformations. The conformational flexibility of this kink therefore appears to be functional.

We also found an example of strong correlation between the kink angles of two different helices across all GPCRs. This is consistent with the theory that it is the environment of a helix and not just its sequence which influences kink angle. We have shown this in the context of homologs, but it would also be interesting to see whether a similar effect can be observed in one protein through the course of a molecular dynamics trajectory.

Kinks are one of the possible deformations of a helix; others such as bulges and twisting may be equally functionally relevant. In the future, the examination of all distortions within a single framework could improve our understanding of the link between structure and function.

Therefore, our investigation has shown significant and widespread variation of kink conformations which could point to their flexibility, and our results support the belief that kinks are important for functional changes of conformation.

## Supporting Information

S1 Document Supplementary figures and legends. (PDF)

S1 Data Data package of results. (GZ)
