# Supplementary material for: Examining the Conservation of Kinks in Alpha Helices
Source: PLoS One. 2016 Jun 17;11(6):e0157553. doi: 10.1371/journal.pone.0157553 (PMC4912094; doi:10.1371/journal.pone.0157553)
Supplement: S1 Document — (PDF) [file pone.0157553.s001.pdf]

Supporting Information

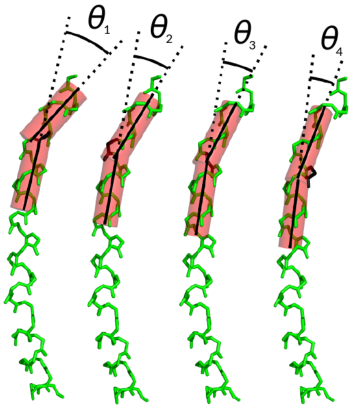

Figure A: Angle measurement by Kink Finder. Cylinders, shown in red, are fitted to each six-residue segment of the helix. Angles θ_1, θ_2... are measured between the axes of adjacent cylinders, and allocated to the last residue of the first segment, shown in black. In this way an angle is assigned to every residue in the helix except the first five and the last six. Adapted from [1].

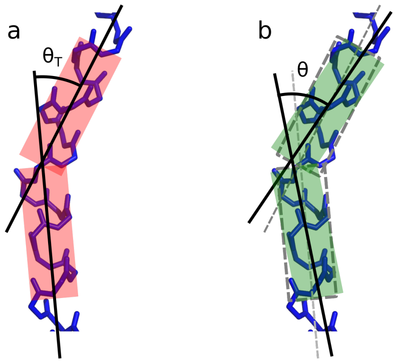

Figure B: **Relating angle error to goodness of fit.** (a) Example 'ideal' kink, with low r_n and r_c. The true angle (θ_T) is the angle between the two fitted axes. (b) Cylinders are rotated (in green) from their fitted positions (dashed lines), and a measured angle (θ) is calculated. r_n and r_c are calculated from the rotated cylinders (green). Carrying out this rotation many times provides the data for Figure C.

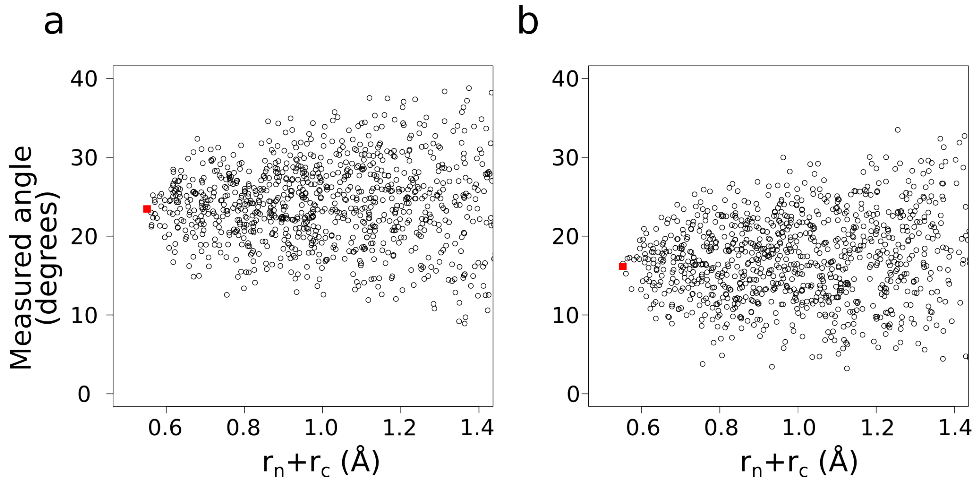

Figure C: Measured angle against goodness of fit ($r_n + r_c$) for two kinks. (a) At residue 255 in chain A of protein 1PB2. (b) At residue 259 in chain A of protein 1Y2L. The red squares indicate the angle and $r_n + r_c$ for the optimum cylinder fits.

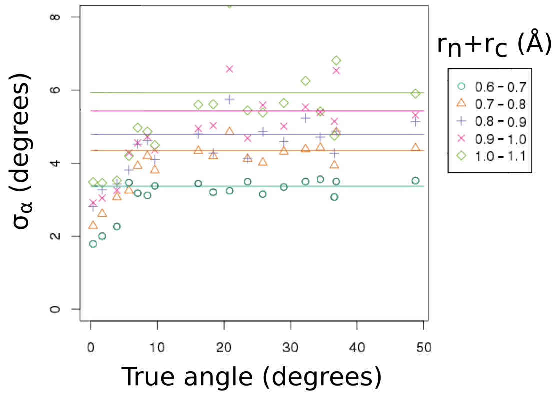

Figure D: The standard deviation, σ_α, of α (measured angle - true angle) for bins of $r_n + r_c$ (y-axis) are shown for 18 ideal kinks, plotted against their true angle as determined by the optimised cylinder fits. The standard deviation of α for a given range of $r_n + r_c$ is constant for angles above 10°. Horizontal lines are fitted to the points where true angle $> 10°$ for each range of $r_n + r_c$.

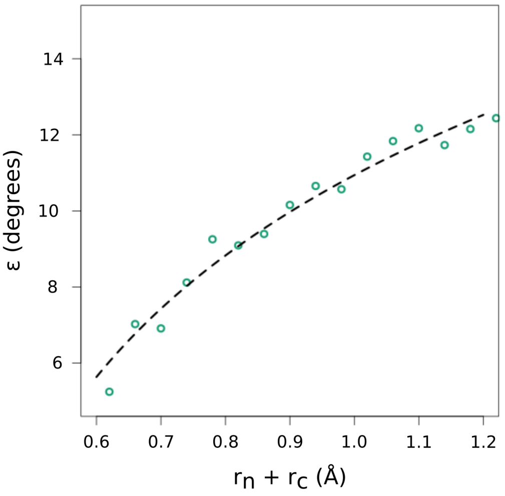

Figure E: The error, ε, for a range of values of $r_n + r_c$ (quality of fit), where ε represents the size of the 95% confidence interval of angle error. For the combined data from all 12 kinks with angles $\geq 12°$, the angle errors are binned by their $r_n + r_c$ values. The value at the 95th percentile of $|\alpha|$ (where α is measured angle - true angle) is taken as the value of ε for each $r_n + r_c$ bin (green points). A log plot is fitted to the values between 0.6 and 1.0 (dashed black line).

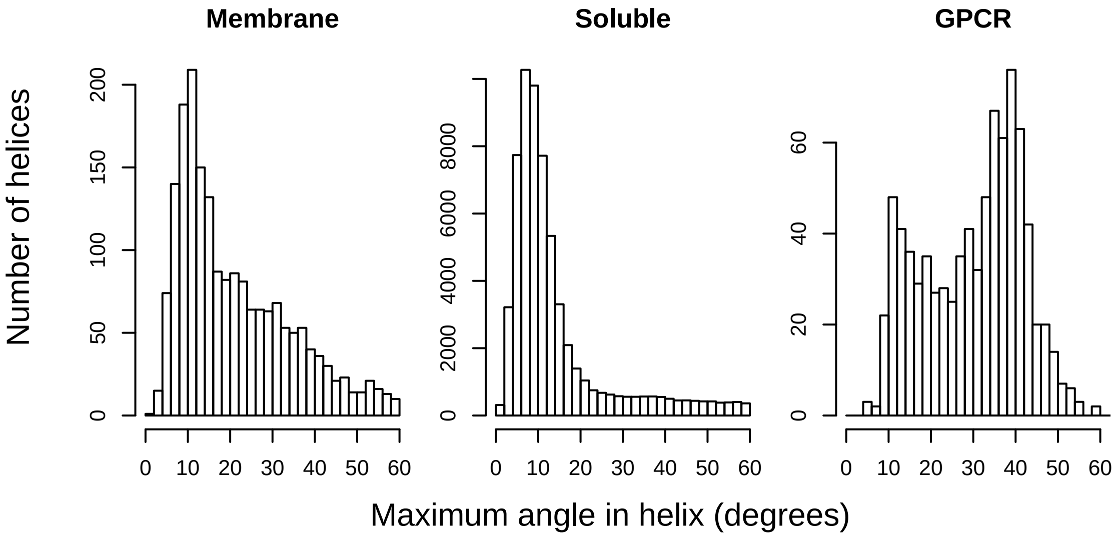

Figure F: The distribution of maximum angles measured by Kink Finder in helices from the membrane, soluble and GPCR data sets.

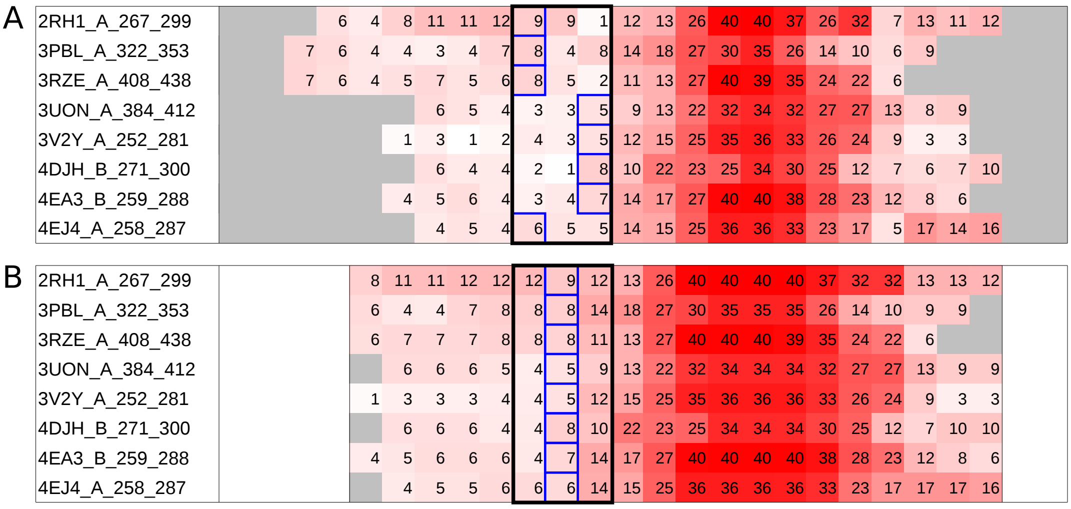

Figure G: **A)** Angle measurements in degrees at each residue of every helix in an example family, in the positions those residues were located when aligned by MAMMOTH-Mult. **B)** Smoothed angles produced by taking the maximum angle in a window of one residue either side of the position.

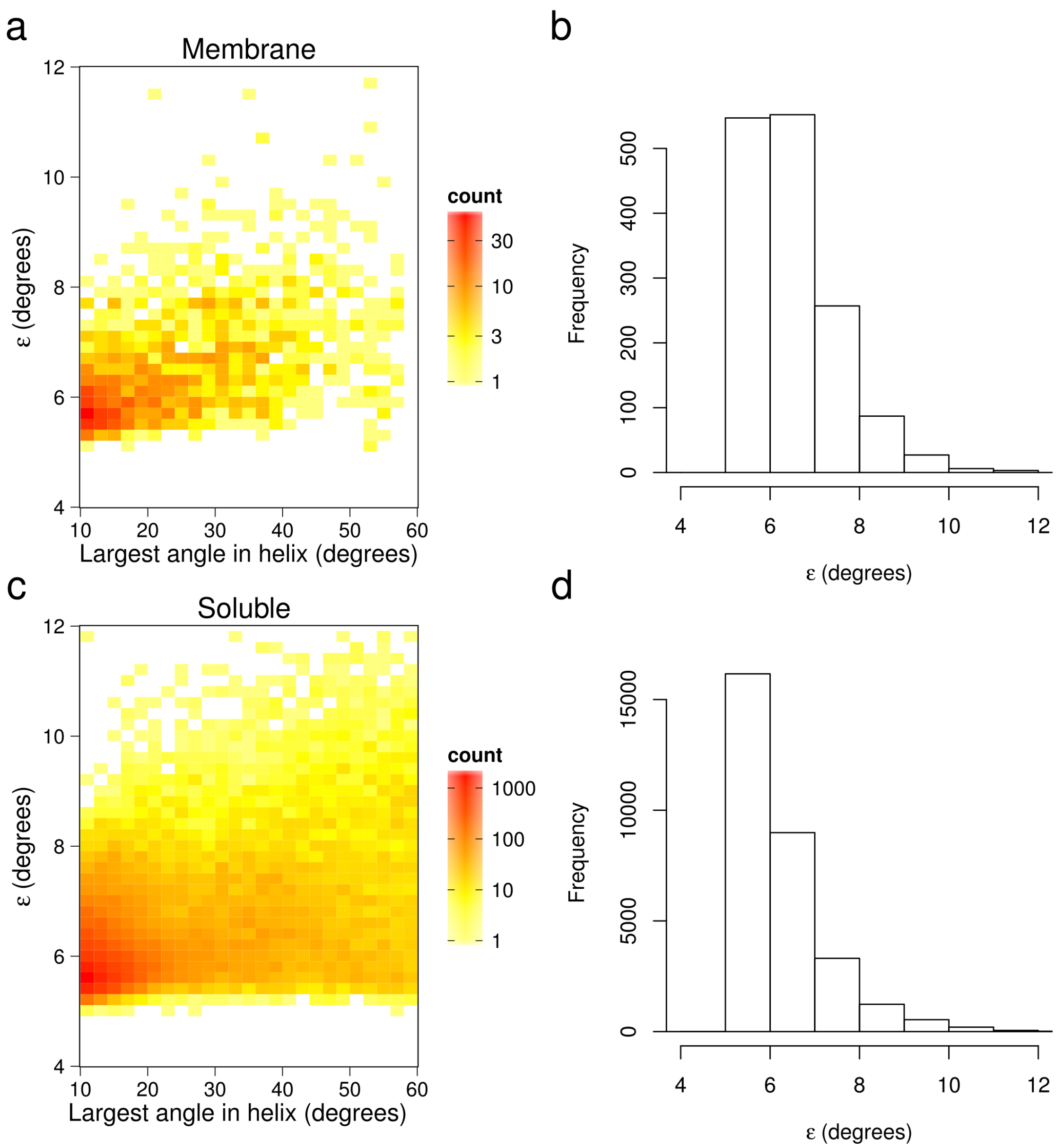

Figure H: **The error, ε, for the maximum kink angles in the membrane (a and b) and soluble (c and d) helices**. Helices with maximum angle $\leq 10°$ are not included. (a) and (c) Heat map showing the variation of ε with angle. Coloured using a log scale. (b) and (d) Histogram of ε.

Non-redundant datasets, resolution < 5 Å and R < 0.4

	Membrane					Soluble				
	CK	CS	NC	other	total	CK	CS	NC	other	total
All	1189	1806	789	320	4104	40190	481388	88390	19556	629524
%	29.0	44.0	19.2	7.8	100.0	6.4	76.5	14.0	3.1	100.0
a) PP	592	36	78	74	780	8457	157	2351	486	11451
P-	167	112	315	46	640	5642	3180	35662	2605	47089
-P	179	10	52	32	273	5040	390	1930	285	7645
- -	251	1648	344	168	2411	21051	477661	48447	16180	563339
b) PP	49.8	2.0	9.9	23.1		21.0	0.0	2.7	2.5	
P-	14.0	6.2	39.9	14.4		14.0	0.7	40.3	13.3	
-P	15.1	0.6	6.6	10.0		12.5	0.1	2.2	1.5	
- -	21.1	91.3	43.6	52.5		52.4	99.2	54.8	82.7	
c) PP	14.4	0.9	1.9	1.8	19.0	1.3	0.0	0.4	0.1	1.8
P-	4.1	2.7	7.7	1.1	15.6	0.9	0.5	5.7	0.4	7.5
-P	4.4	0.2	1.3	0.8	6.7	0.8	0.1	0.3	0.0	1.2
- -	6.1	40.2	8.4	4.1	58.7	3.3	75.9	7.7	2.6	89.5

High quality non-redundant dataset, resolution < 2 Å and R < 0.2

	Soluble				
	CK	CS	NC	other	total
All	12622	136141	27894	5972	182629
%	6.9	74.5	15.3	3.3	100.0
a) PP	2647	36	734	112	3529
P-	1685	1156	11258	796	14895
-P	1483	94	612	81	2270
- -	6807	134855	15290	4983	161935
b) PP	21.0	0.0	2.6	1.9	
P-	13.3	0.8	40.4	13.3	
-P	11.7	0.1	2.2	1.4	
- -	53.9	99.1	54.8	83.4	
c) PP	1.4	0.0	0.4	0.1	1.9
P-	0.9	0.6	6.2	0.4	8.2
-P	0.8	0.1	0.3	0.0	1.2
- -	3.7	73.8	8.4	2.7	88.7

Table A: The number of aligned helix pairs in each class, and occurrence of proline at the position with the largest angle or in the four following residues. The helix pair classes CK, CS, NC and other are defined in the Results. PP: proline in both helices; P-: proline in the helix with the larger kink angle; -P: proline in the helix with the smaller kink angle; - -: proline in neither helix. a) the frequency of each type in each class b) the frequency of each type as a percentage of the pairs in that class c) the frequency of each type as a percentage of the total number of pairs.

Mem/ Sol	Resolution cutoff (Å)	R factor cutoff	PISCES cull %SID	Proline kinks included	Number of helix pairs in dataset	Correlation coefficient with angle difference			Partial Correlation coefficient with angle difference					
						Helix	Neigh	Global	Helix (controlling for Global)	Global (controlling for Helix)	Helix (controlling for Neigh)	Neigh (controlling for Helix)	Neigh (controlling for Global)	Global (controlling for Neigh)
M	5	0.4	80	Yes	4105	-0.278	-0.290	-0.265	-0.134	-0.101	-0.110	-0.140	-0.132	-0.048
M	5	0.4	80	No	2412	-0.228	-0.225	-0.214	-0.107	-0.070	-0.103	-0.095	-0.084	-0.044
S	5	0.4	80	Yes	630333	-0.127	-0.115	-0.098	-0.087	-0.031	-0.079	-0.057	-0.063	-0.019
S	5	0.4	80	No	563959	-0.096	-0.101	-0.084	-0.058	-0.035	-0.050	-0.059	-0.058	-0.013
S	2	0.2	80	Yes	182860	-0.121	-0.107	-0.093	-0.083	-0.030	-0.077	-0.052	-0.057	-0.019
S	2	0.2	80	No	162111	-0.088	-0.094	-0.077	-0.054	-0.033	-0.046	-0.056	-0.054	-0.010

Table B: Table of Spearman's rank correlation coefficients between angle difference ($|\theta_{max} - \theta_{min}|$) and each measure of sequence conservation. The three measures are helix sequence identity (Helix), neighbouring sequence identity (Neigh, see Methods), and global sequence identity (Global). Partial correlation coefficients are also given for the three types of sequence identity, using each of the other measures of sequence identity as a controlling variable. Results are shown for each non-redundant data set, and also for each of these sets after any helix pair with proline at the kink site was removed.

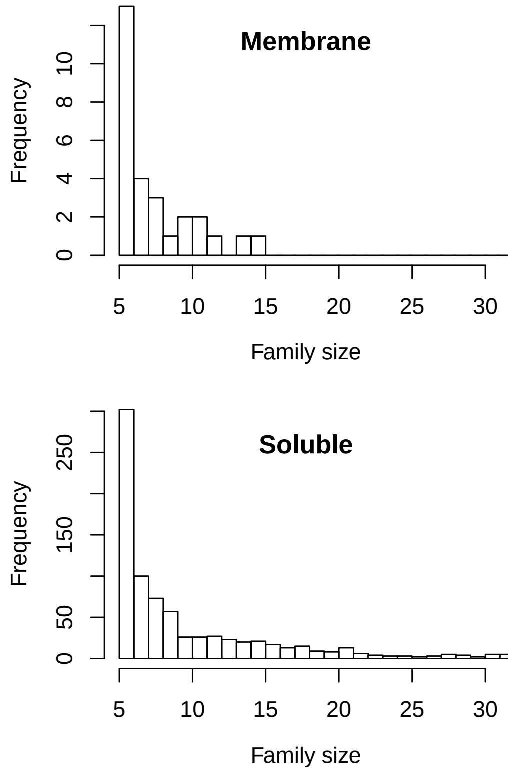

Figure I: Distribution of sizes of families of at least 5 members.

A

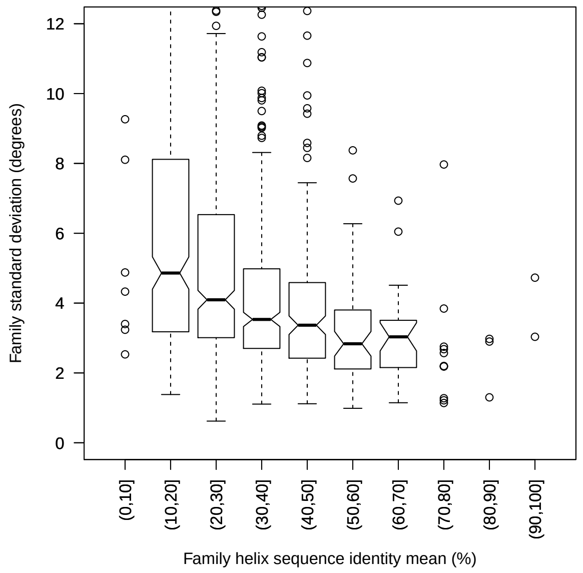

B

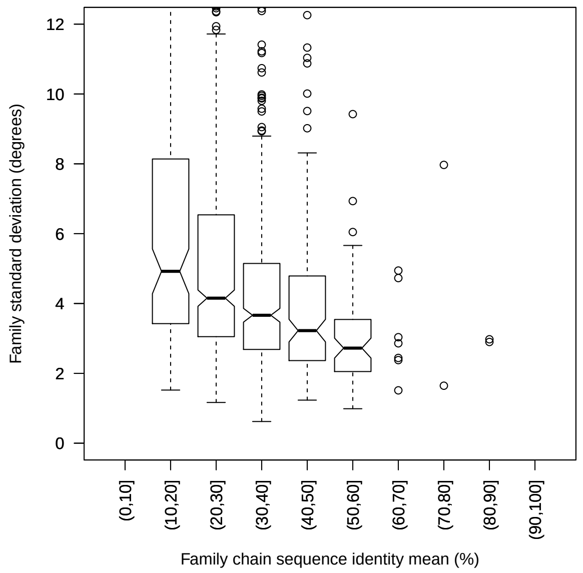

Figure J: The standard deviation of angles at the most disrupted site in a family plotted against the family mean sequence identity between A) all pairs of homologous helix sequences and B) all pairs of homologous chain sequences. Data from the non-redundant membrane and soluble protein sets combined, as the membrane set is small but appears to show a similar distribution to the soluble set (Figure K).

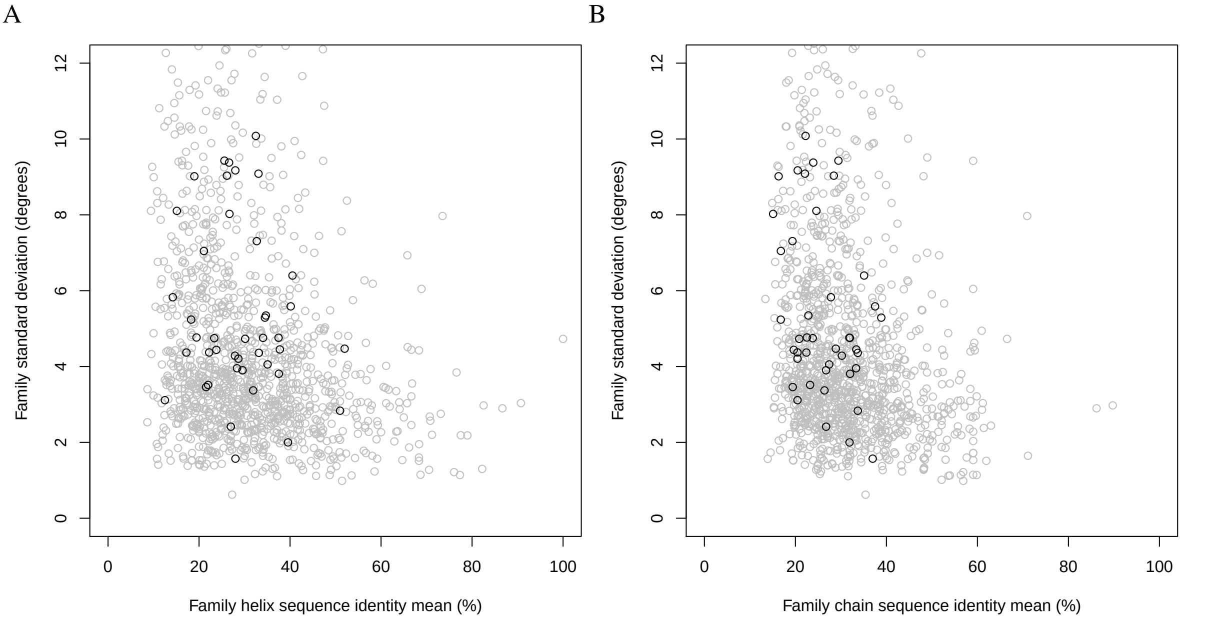

Figure K: The standard deviation of angles at the most disrupted site in a family plotted against the family mean sequence identity between A) all pairs of homologous helix sequences and B) all pairs of homologous chain sequences. Data from the non-redundant membrane (black) and soluble (grey) protein sets.

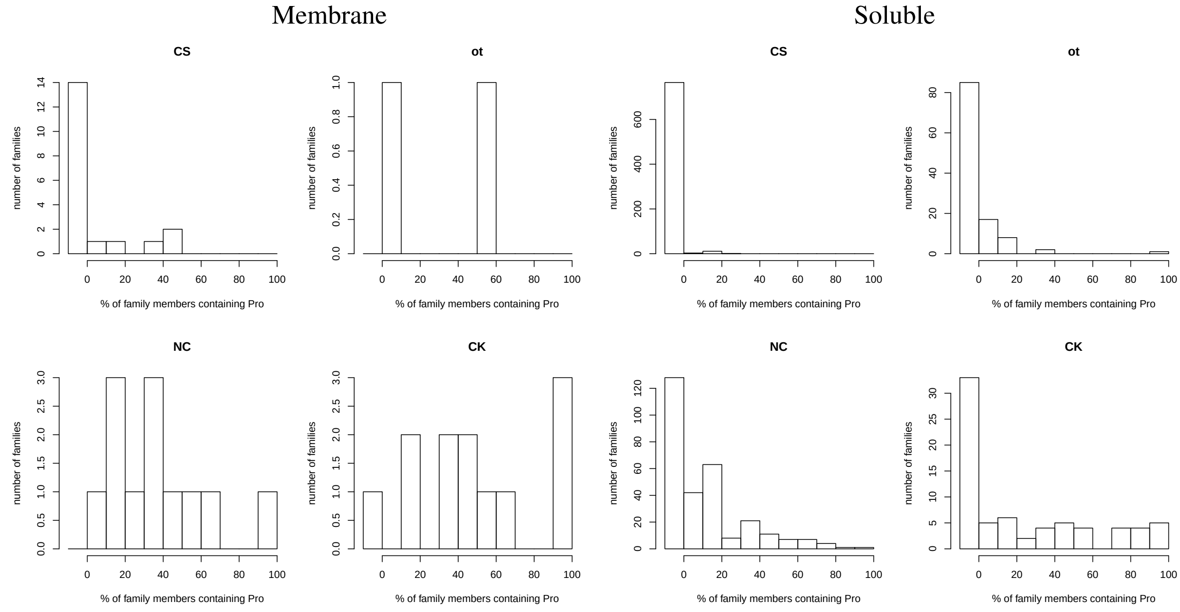

Figure L: The percentage of family members containing proline at the most disrupted site in the helix family or in the four following residues, broken down by family class. The left-most bar represents families with no proline. CS: Conserved Straight, ot: Other, NC: Not Conserved, CK: Conserved Kinked.

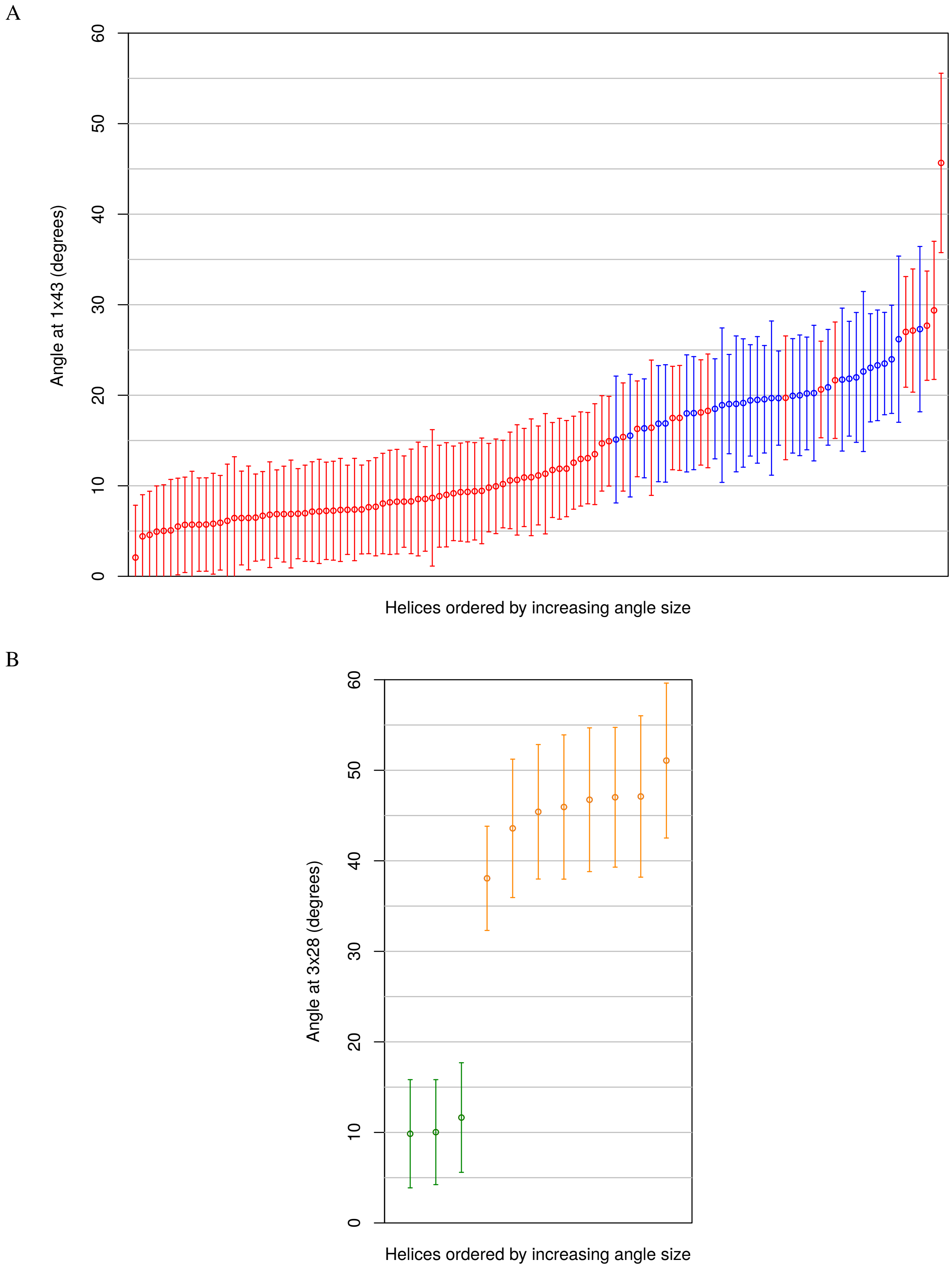

Figure M: Angles from the histograms in Figure 7 ordered by magnitude and shown with their estimated 95% confidence intervals ($\pm\varepsilon$). **A)** Angles at position 1x43 in all GPCR structures. Angles from rhodopsin structures are shown in blue; angles from all other structures in red. **B)** Angles at position 3x28 in the human adenosine A_{2A} receptor. Agonist-bound receptors are shown in green (n=3); antagonist-bound receptors in orange (n=8).

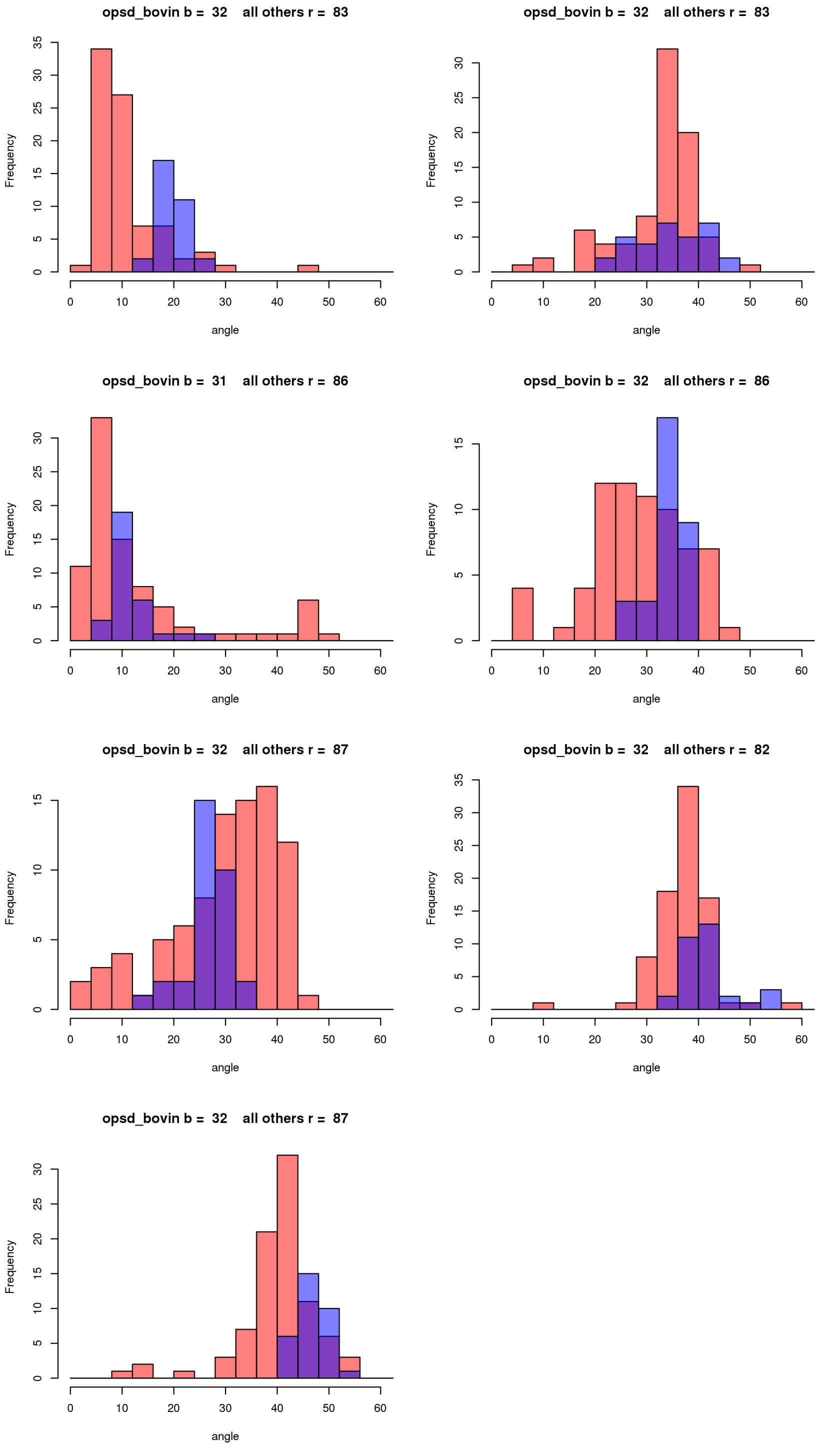

Figure N: Angle distribution at the maximum site of disruption for each TMH of the GPCR family. Angles from rhodopsin structures are shown in blue; angles from all other structures in red.

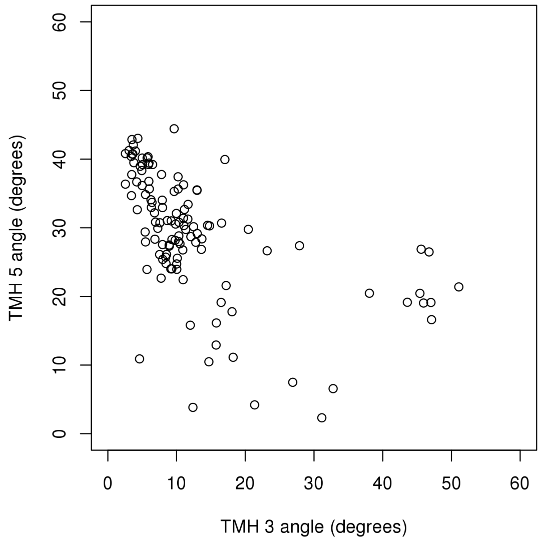

Figure O: Correlation between the magnitude of angles at position 3x28 (TMH 3) and 5x46 (TMH 5) observed in all GPCR structures.
